# Patterns, prevalence and determinants of environmental tobacco smoke exposure among adults in Bangladesh

**DOI:** 10.1016/j.abrep.2018.09.001

**Published:** 2018-09-12

**Authors:** Mohammad Alamgir Kabir, Md. Moyazzem Hossain, Farhana Afrin Duty

**Affiliations:** Department of Statistics, Jahangirnagar University, Savar, Dhaka, Bangladesh

**Keywords:** Environmental tobacco smoke exposure, Equiponderant graphs, Public health, Bangladesh

## Abstract

**Background:**

Exposure to environmental tobacco smoke (ETS) has been suggested as a risk factor for various health problems. Thus, this study examines the patterns and predictors of ETS exposure among adults at home, workplace and public places.

**Methods:**

The dataset covered a nationally representative sample of 9629 respondents extracted from the Global Adult Tobacco Survey. Diamond-shaped equiponderant graphs were used to exhibit the prevalence of ETS. In Logistic regressions, ETS exposure at home, workplace and public places were used as response variables. Demographic and socioeconomic variables, health knowledge about ETS, attitude towards ETS, perception of smoking restrictions were considered as predictors.

**Results:**

Adults in higher age groups and females were less exposed to ETS. Better education, high wealth status, better health knowledge on ETS, practice of no smoking at home, and support smoking restrictions were significantly associated with lower ETS exposure at home. Those residing in rural areas and living with many people together were more likely to be exposed to ETS at home. In contrast with home and workplace exposure, adults with higher education, better wealth status, good knowledge on ETS, and support smoking restrictions experienced a high level of exposure at public places. Interestingly, results suggest that those with high levels of ETS exposure at home and workplace had lower exposure to ETS in public places.

**Conclusions:**

ETS control should not be overlooked in public health policy. Protection from ETS at home is particularly important, given its impact on the attitude towards and awareness about ETS exposure at all places.

## Highlights

• Exposure to environmental tobacco smoke (ETS) has been established as a causal factor for a number of health problems.
• This study examines the patterns and predictors of ETS exposure among adults at home, workplace and public places.
• Diamond-shaped equiponderant graphs were used to exhibit the prevalence of ETS.
• Logistic regressions were estimated with ETS exposure at home, workplace and public places as the response variable.

## 1 Introduction

Environmental tobacco smoke (ETS) exposure is one of the most common preventable health hazards in the community. The estimated attributable deaths due to ETS totaled 603,000, of which 28% were estimated to be children (World Health Organization, 2009a, World Health Organization, 2009b). Although ETS exposure is a well-known risk factor for cancer in adults, there is emerging evidence that it may also be associated with childhood cancers (Boffetta, Tredaniel, & Greco, 2000; Filippini et al., 2002; Krajinovic, Richer, Sinnett, Labuda, & Sinnett, 2000). ETS has been established as a causal risk factor for a number of health problems for women, and adults. In pregnant women, reduced fetal growth, low birth weight, pre-term delivery and sudden infant death were linked to ETS exposure (CEPA, 2005; Filippini et al., 2002). Other associated risks include: spontaneous abortion, intrauterine growth retardation, adverse impacts on cognition and behaviour, allergic sensitization, elevated decreased pulmonary function growth and adverse effects on fertility or fecundity, and elevated risk of stroke (CEPA, 2005). Smoking tobacco especially cigarette/*bidis*^1^ is the principal source of exposure of nonsmokers or smokers to tobacco smoke. The burning cigarette produces smoke primarily in the form of mainstream smoke (MS), that is the smoke inhaled by the smoker during puffing and side stream smoke (SS), that is the smoke released by the smoldering cigarette while not being actively smoked (Eriksen, Mackay, & Ross, 2012). Nonsmokers or smokers are exposed to the combination of diluted SS that is released from the cigarette's burning end and the MS exhaled by the active smoker (First, 1985). This mixture of diluted SS and exhaled MS has been referred to as ETS. Exposure to ETS is also commonly referred to as passive or involuntary smoking. Over 3000 different chemicals, including irritant gases, carcinogens and fine particles are contained in tobacco smoke (World Health Organization, 2009a, World Health Organization, 2009b). Nonsmokers or smokers who live or work with a smoker generally have the greatest exposure to ETS. Although ETS in public places is important as a nuisance, it usually contributes only a small amount to personal ETS exposure (World Health Organization, 2009a, World Health Organization, 2009b).

In addition to a large and growing health burden, ETS exposure also imposes economic burdens on individuals and countries, both for the costs of direct health care as well as indirect costs from reduced productivity (World Health Organization, 2009a, World Health Organization, 2009b). Literature showed socio-economic and demographic factors, and knowledge, attitude and perception (KAP) towards ETS to significantly influenced the exposure level (Abdullah et al., 2011; Bolte et al., 2009; Hyland et al., 2009; Sims et al., 2010; Akhtar, Currie, Currie, & Haw, 2007; Rachiotis et al., 2010; Rudatsikira, Siziya, Dondog, & Muula, 2007; Mei et al., 2009; Oberg, Jaakkola, Woodward, Peruga, & Pruss-Ustun, 2011; Mak, Loke, Abdullah, & Lam, 2008; Liu et al., 2008; Chen et al., 2009).

Like direct smoking, ETS was linked to enormous health problems among adults (Eriksen et al., 2012; Oberg et al., 2011; World Health Organization, 2010a, World Health Organization, 2010b, World Health Organization, 2010c). Evidence shows that the ETS problem is also serious in Bangladesh compared to developed countries and this is due to population density, lower level of knowledge and awareness, lack of strict public law enforcement (Oberg et al., 2011; World Health Organization, 2010a, World Health Organization, 2010b, World Health Organization, 2010c). Numerous studies have been conducted on ETS and their adverse health effects in many developed countries and some middle-income countries. However, comprehensive research on developing countries where their consequences are serious lags behind. Research on ETS and its influencing factors in Bangladesh are scarce and limited to micro level data. Thus, this study examines the patterns and predictors of ETS exposure among adults at home, workplace and public places. Moreover, the factors identified in this study based on nationally representative data will help to fill the research gap and also offer helpful insights for the design and implementation of smoke free environment in Bangladesh and can replicate to other developing countries for policy action.

## 2 Materials and methods

### 2.1 The data and sampling

The data for this study were obtained from Global Adult Tobacco Survey-2009. The detailed methodology of data collection, sampling procedure, questionnaires and relevant information were reported in GATS Bangladesh report (World Health Organization, 2010a, World Health Organization, 2010b, World Health Organization, 2010c). Briefly, based on the sampling frame from Bangladesh Bureau of Statistics, the implementing agency of Bangladesh population census in 2001, the GATS was a three-stage stratified cluster sample of households. In the first stage, 400 primary sampling units (PSUs) (200 from rural and another 200 from urban areas) were selected with probability proportional to size. In the second stage, a random selection of one secondary sampling unit (SSU) per selected PSU was done. The SSUs were based upon the enumeration areas (EAs) from Bangladesh Agricultural Census (2008). Each EA's consisted of 200 households in rural areas and 300 households in urban areas. In the third stage, households were selected systematically within the listed households from a selected SSU (an average of 28 households to produce equal male and female households). The sample consisted of 11,200 non-institutional households from all 6 administrative divisions covering 95.5% of the total population. One respondent was randomly selected for interview from each selected eligible household to participate in the survey. About 10,751 (96.0%) households and 9629 (86.0%) individuals successfully completed the interview. The sample design for GATS Bangladesh provides cross-sectional estimates for the country as a whole as well as by urban, rural and gender.

### 2.2 The tools of data collection

GATS in Bangladesh used two types of questionnaires, namely, household and individual. The questionnaires were based on GATS core and optional questions. The Ministry of Health & Family Welfare of Bangladesh with the consultation of local agencies (NIPSOM, NIPORT, BBS) and international collaborators such as WHO South East Asia Regional Office and Centers for Disease Control and Prevention conducted the survey. The survey used electronic system (handheld computer) that facilitated the complex skip pattern used in the GATS questionnaire, as well as some in-built validity checks on questions during the data collection. The main steps involved in quality control checks were: version checking for household and individual questionnaires, checking date and time, skipping patterns and validation checks. To improve representativeness of the sample in terms of the size, distribution and characteristics of the population, the data were suitably weighted. The weights were derived from design weight, household and individual response rates. The detailed weighting procedure can be found in Global Tobacco Surveillance System (GTSS), Global Adult Tobacco Survey (GATS): Sample Design Manual & Sample Weights Manual (World Health Organization, 2010a, World Health Organization, 2010b, World Health Organization, 2010c).

### 2.3 The dependent and predictor variables

ETS at different settings, namely, home, workplace, and public places was considered as the response variable. Exposure at other places was excluded from bivariate and multivariate analysis due to small number of cases. Following the theory and literature on ETS exposure, and the nature of supporting data, predictors namely, age, gender, household members, residence, education, wealth index, general and specific health knowledge about ETS exposure, attitude about ETS at home and workplace, and perception of smoking restrictions at some places were selected for current study. The detailed of the variables and their coding for analysis are given in Table 1.

**Table 1:** Variables for this Study and their Coding for Analysis.

Response variable: Environmental tobacco smoke (ETS) exposure		
Variables name	Question asked in the survey	Coding for analysis
Exposed at home	How often does anyone smoke inside your home? Options: 1 = daily; 2 = weekly; 3 = monthly; 4 = less than monthly; 5 = never	1 = yes (option 1 to 3) 0 = no (option 4 & 5)
Exposed at workplace	During the past 30 days, did anyone smoke in indoor areas where you work? Options: 1 = yes; 2 = no	1 = yes (option 1) 0 = no (option 2)
Exposed at public places	Did anyone smoke inside of any …? that you visited in the past 30 days? Options for all type of places	1 = yes (option 1) 0 = no (option 2)
Exposed at Government buildings or offices.		1 = yes (option 1); 0 = no (option 2)
Exposed at health care facilities.		1 = yes (option 1); 0 = no (option 2)
Exposed at restaurants.		1 = yes (option 1); 0 = no (option 2)
Exposed public transportation.		1 = yes (option 1); 0 = no (option 2)


The selected variables as predictors		
Variables name	Question asked in the survey	Coding for analysis
Age in years	How old are you? Open ended question	15–24, 25–44, 45–59, 60+
Gender	Record gender from observation	1 = male, 2 = female
No. of persons in household,	In total how many persons live in this household? Open ended question	1–2,3-4,5-9,10+
Residence	What is the place of residence?	1 = urban, 2 = rural
Education	Highest level of education? 1 = no formal education; 2 = less than primary school completed; 3 = primary school completed; 4 = less than secondary school completed; 5 = secondary school completed; 6 = high school completed; 7 = college or university completed; 8 = post graduate degree completed; 77 = don't know	1 = no formal education (option 1 & option 77); 2 = less than primary; 3 = primary completed 4 = less than secondary; 5 = secondary & above (option 5 to 8)
Wealth index	Household or any person in the household has: a. electricity b. flush toilet c. fixed telephone d. cell phone e. television f. radio g. refrigerator h. car i. motorcycle j. washing machine k. bicycle l. sewing machine m. wardrobe n. table o. bed or cot p. chair q. watch.	1st quintile: lowest 2nd quintile: low 3rd quintile: middle 4th quintile: high 5th quintile: highest
General health knowledge about ETS	Based on what you know or believe, does ETS cause serious illness in non-smokers? 1 = yes; 2 = no; 7 = don't know	1 = yes (option 1) 2 = no (option 2 and 7)
Specific health knowledge about ETS exposure	Based on what you know or believe does ETS cause any of the following: a. heart disease in adults? b. lung cancer in adults? c. lung illness in children? For all three type of questions: 1 = yes; 2 = no; 7 = don't know	0 = No knowledge (answer no questions correctly); 1 = Some knowledge (answer any one or two questions correctly); 2 = Good knowledge (answer all questions correctly)
Attitude about ETS (at home)	Smoking policy inside your home? Options: 1 = allowed; 2 = not allowed, but exception; 3 = never allowed; 4 = no rules; 7 = don't know	1 = smoking allowed; 2 = not allowed; 3 = no rules/policy
Attitude about ETS (at workplace)	Indoor smoking policy where you work? Options: 1 = allowed anywhere; 2 = allowed only in some indoor areas; 3 = not allowed in any indoor areas; 4 = there is no policy; 7 = don't know	1 = smoking allowed; 2 = not allowed; 3 = no rules/policy
Perception to smoking restrictions at other places	If you think smoking should or should not be allowed in indoor areas? Workplaces? 2. Restaurants? 3. Universities? For all three type of questions: 1 = should be allowed; 2 = should not be allowed; 7 = don't know	0 = no support (all answers for allowed); 1 = moderate support (answer any 1 or 2 in favour of restrictions); 2 = strong support (answer all for restrictions)
For tobacco consumption	Do you currently smoke tobacco or using smokeless tobacco? Options: 1 = daily; 2 = less than daily; 3 = not at all; 7 = don't know	For both products: 0 = not at all; 1 = daily; and 2 = less than daily

### 2.4 Statistical analyses

Statistical analyses were performed using SPSS version 20 (SPSS Inc., Chicago, IL). Frequency runs were generated to compute the prevalence of ETS at three settings. Diamond-shaped equiponderant graphs were used to exhibit the prevalence of ETS (Li, Buechner, Tarwater, & Muñoz, 2003). Bivariable analyses using cross tabulations were also performed to obtain the prevalence of ETS for various categories of the selected variables and to identify significant determinants using the Pearson's Chi-square (χ^2^) test (Chan, 2003). Determinants that significantly explain ETS at three settings were entered into the logistic regressions for multivariable analyses (Chan, 2004). We utilized three binary logistic regression models separately for three different settings (Model A: exposed at home, Model B: exposed at workplaces, and Model C: exposed at public places).

The general logistic regression model is given by:

$\mathrm{Pr}\;\left(Y_{i}=1\right)=\frac{\exp\;\left(X_{i}\beta \right)}{1+\exp\;\left(X_{i}\beta \right)}$where *Y*_*i*_ is a binary variable that takes a value of ‘1’ if the respondent is exposed ETS and ‘0’ otherwise, *X*_*i*_ is a vector of independent variables and *β* is a vector of unknown parameters.

The estimated form of regression is as:

(1) $$\ln\;\left[\frac{P_{i}}{1-P_{i}}\right]=\beta_{0}+\beta_{1}X_{1}+\ldots +\beta_{k}X_{k}$$

The odds ratio (OR) in favour of *Y*_*i*_ = 1 together with its 95% confidence interval (CI) were computed for *X*_1_, *X*_2_, …, *X*_*k*_ to indicate how many times the group of interest is more likely to be exposed ETS compared to the reference group.

To examine the association between tobacco consumption (TC) and ETS after controlling for predictors used earlier, another model is as:

(2) $$\mathrm{Pr}\;\left(Y_{i}=1\right)=\mu +\alpha X_{i}+\beta_{1}Z_{1}+\beta_{2}Z_{2}+\ldots +\beta_{k}Z_{k}$$

where *Y*_*i*_ = 1 if respondent-*i* is exposed ETS and 0 otherwise, *X*_*i*_ = 1 if respondent-*i* is a tobacco users and 0 otherwise, and *Z*_1_, *Z*_2_, …, *Z*_*k*_ are variables used earlier as predictors that affect ETS. For instance, *Y*_*i*_ was assigned a value of 1 if the respondent is exposed ETS. The TC variable *X*_*i*_ was assigned a value of 1 if the respondent were current tobacco users. For comparison purposes, the regression was also estimated separately for the two different forms of TC namely, smoked tobacco and smokeless tobacco products. The odds ratios and their 95% confidence interval for examining the association between TC and ETS were computed after controlling for other variables.

## 3 Results

### 3.1 Basic profile of the respondents

>40% of the respondent were 25–44 years. The average age was about 36 years. The male-female ratio was almost same (50.3% vs. 49.7%). About 43% adults had household size of 4–5 members with average family size was 5 members. About 74% adults lived in rural area (Table 2). Majority of the respondents (about 36%) have no formal education. Only 8% adults completed higher education (college or university or higher). About 42% came from lowest two wealth quintiles whereas, 38% came from highest two wealth quintiles.

**Table 2:** Distribution of adults by socio-demographic characteristics.

Variables or predictors	%
Age in years (mean ± SD)	35.84 ± 15.96
15–24	29.5
25–44	43.1
45–59	17.2
60 and above	10.2
Gender	
Female	50.3
Male	49.7
Household members(mean ± SD)	5.07 **±** 2.39
1–3	24.0
4–5	42.7
6–8	26.0
9 and above	7.2
Place of residence	
Urban	26.2
Rural	73.8
Educational attainment	
No formal education	36.0
Less than primary to primary completed	27.7
Less than secondary to secondary completed	28.3
Higher education	8.0
Wealth index (asset quintile)	
1st quintile	18.7
2nd quintile	23.3
3rd quintile	20.2
4th quintile	22.8
5th quintile	15.0

### 3.2 Diamond-shaped equiponderant graph of ETS exposure

**Fig. 1:**
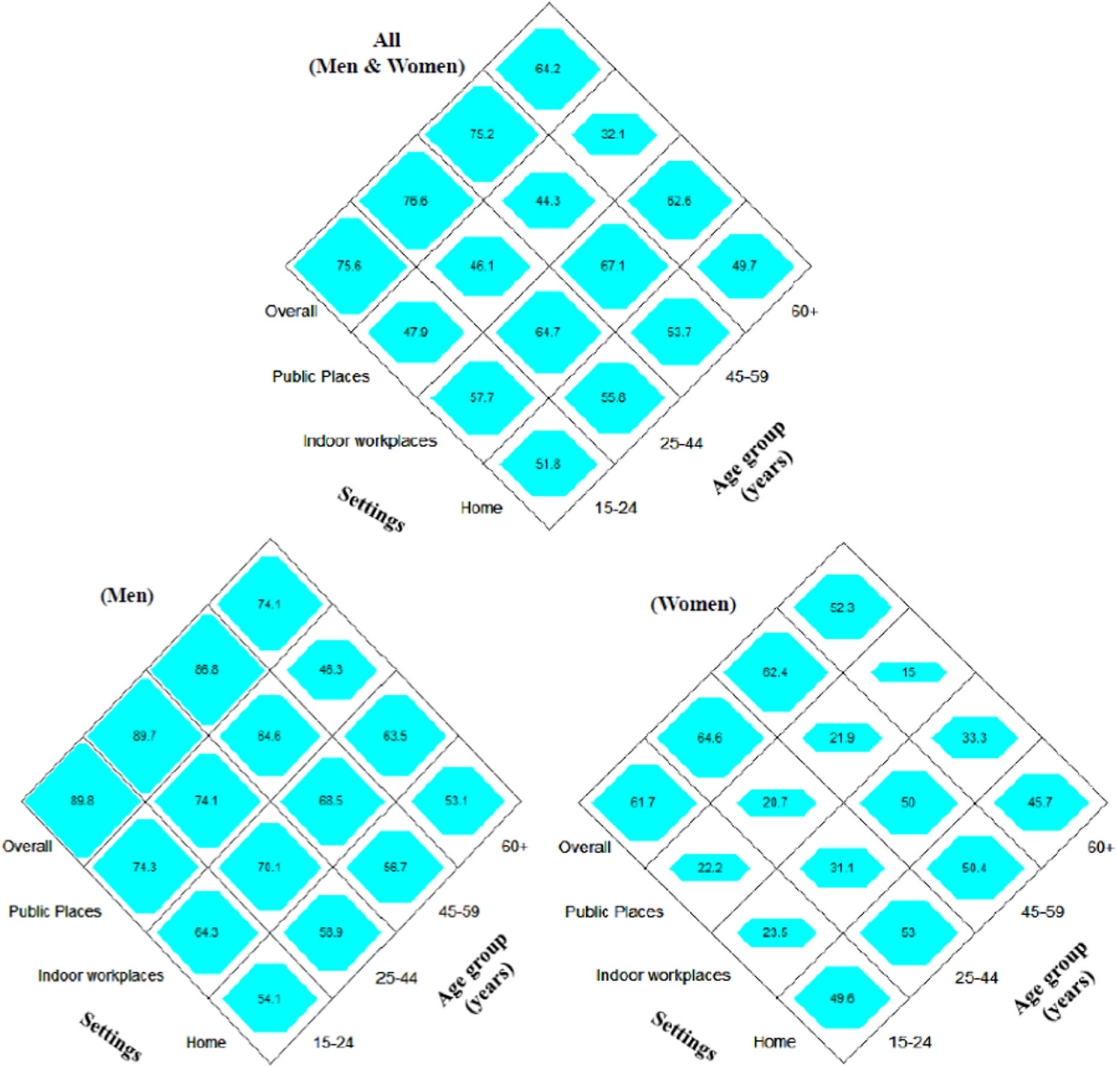
Prevalence of ETS Exposure among Men, Women and Both by Age Groups and Settings.

The overall prevalence rate of ETS exposure and the prevalence rates of ETS in different settings by age groups were calculated for gender. The adults aged 25–44 years old were more exposed by ETS (76.5%) than other age groups in all settings together. This age group was also exposed more in the home (56.6%) than other groups. However, adults aged 15–24 years and aged 45–59 years old were more exposed in public places (47.9%) and indoor workplaces (67.1%) respectively than other groups. Adults aged 60 years and above was less exposed than other groups in all settings. The sex differentials in exposure level were remarkably higher among males than females in all settings. However, in the home, gaps in exposure level between males and females were relatively narrower in comparison to other settings (Fig. 1).

### 3.3 Factors associated with ETS exposure

Table 3 shows the prevalence of ETS exposure in different settings (home, workplace, and public places) by various indicators including socio-demographic factors, health knowledge, perception and attitude about smoking. In bivariate analysis, age, gender, educational level, general and specific health knowledge on ETS, and attitudes to ETS at home were significantly (p < 0.001) associated with ETS exposure in all the three settings. Place of residence, and wealth index were also significant determinants (p < 0.001 to p < 0.05) in three settings. Perception to smoking restrictions at some places (say workplaces, restaurants, universities, etc.) were significant (p < 0.001) for ETS exposure at home and at public places but nonsignificant at workplace. Number of persons in the household and attitude about ETS at workplaces that was only used in Model A, and Model B were statistically significant (p < 0.001) for ETS exposure in both settings.

**Table 3:** ETS exposure in different settings by selected variables.

Variables	ETS exposure in different settings					
	Model A		Model B		Model C	
	% yes	χ^2^ (P)	% yes	χ^2^ (P)	% yes	χ^2^ (P)
Age (in years)						
15–24	51.8		57.7		47.9	
25–44	55.8	18.3	64.7	17.90	46.1	77.13
45–59	53.7	(<0.001)	67.1	(<0.001)	44.3	(<0.001)
60+	49.7		62.6		32.1	

Gender						
Male	56.4	29.15	67.8	122.78	69.3	2268.14
Female	50.9	(<0.001)	30.5	(<0.001)	20.8	(<0.001)

No of persons in household						
1–2 persons	47.7					
3–4 persons	51.1	43.82	–	–	–	–
5–9 persons	55.8	(<0.001)				
10 or more persons	62.4					

Place of residence						
Urban	43.4	143.89	58.8	9.97	46.4	4.70
Rural	57.3	(<0.001)	66.0	(0.002)	44.4	(0.049)

Educational level						
No formal education	62.9		71.1		38.7	
Less than primary	59.2		70.5		47.2	
Primary completed	50.8	344.53	66.7	59.03	40.8	164.94
Less than secondary	47.9	(<0.001)	64.2	(<0.001)	46.9	(<0.001)
Secondary & above	36.0		49.4		58.0	

Wealth index						
Lowest (1st quintile)	66.3		66.1		37.9	
Low (2nd quintile)	58.8		67.6		42.7	
Middle (3rd quintile)	53.3	345.57	62.2	12.65	47.0	63.4
High (4th quintile)	50.4	(<0.001)	63.1	(0.048)	48.4	(<0.001)
Highest (5th quintile)	35.1		59.0		49.0	

GHK on ETS exposure						
Yes	52.8	39.43	62.6	17.20	46.9	214.93
No	65.7	(<0.001)	85.7	(<0.001)	16.8	(<0.001)

Specific HK on ETS						
No knowledge	63.1		72.8		20.5	
Some knowledge	53.4	50.37	53.8	15.7	36.2	410.8
Good knowledge	52.1	(<0.001)	63.2	(<0.001)	50.3	(<0.001)

Attitude on ETS (home)						
Smoking allowed	93.9		67.8		42.4	
Not allowed	18.4	3870.0	57.3	31.38	47.0	15.50
No rules or policy	68.3	(<0.001)	71.1	(<0.001)	44.1	(<0.001)

Attitude on ETS (workplace)						
Smoking allowed			89.4			
Not allowed	–	–	21.9	684.4	–	–
No rules or policy			75.6	(<0.001)		

Perception of SR at SP						
No supports	62.5		100.0		9.6	
Moderate supports	73.0	51.35	73.2	4.38	47.7	53.68
Strong supports	52.9	(<0.001)	62.6	(0.112)	45.2	(<0.001)

GHK-general health knowledge, SR-smoking restrictions, SP-some places (workplaces, restaurants, universities, etc.), χ^2^ **=** Pearson Chi-square test, P = *p*-value; Model A: exposed at home, Model B: exposed at workplace, and Model C: exposed at public places.

For Model A, the results showed that adults aged 45–59 and 60+ years old had significantly (OR = 0.76, 95% CI = 0.63–0.91; OR = 0.54, 95% CI = 0.44–0.67) lower odds of ETS exposure at home compared to reference category of 15–24 years old. Similarly, females had 56% lower likelihood of ETS exposure than males. Higher number of persons in a household significantly (p < 0.001 to p < 0.05) increased the odds of ETS exposure at home. For example, there was 1.2, 1.5 and 2.4 times higher likelihood of ETS exposure at home if the household had 3–4, 5–9, and 10 or more persons respectively compared with reference category of living 1–2 persons. The respondents from rural area had 1.4 times higher odds of ETS exposure than urban residents. The respondents with education of completed primary, less than secondary and secondary & above had 30%, 40%, and 50% lower odds to be exposed at home respectively than respondents with no education. Similarly, respondents with low to middle wealth quintiles had significantly (16–19%) lower odds to be exposed to ETS at home compared to lowest wealth quintiles whereas respondents with high (OR = 0.75, 95% CI = 0.63–0.90) and highest (OR = 0.63, 95% CI = 0.50–0.78) wealth quintiles had significantly (p < 0.001) lower likelihood to be exposed at home compared with reference category of lowest wealth index group (Table 4).

**Table 4:** List of covariates adjusted for ETS exposure in different settings.

Variables	ETS exposure in different settings		
	Model A	Model B	Model C
	OR (95% CI)	OR (95% CI)	OR (95% CI)
Age (in years)			
15–24	–	–	–
25–44	1.04 (0.91–1.19)	1.44 (1.06–1.96)***	1.00 (0.89–1.13)
45–59	0.76 (0.63–0.91)***	0.91 (0.61–1.35)	0.84 (0.72–0.97)**
60+	0.54 (0.44–0.67)***	0.65 (0.37–1.13)	0.43 (0.35–0.51)***

Gender			
Male	–	–	–
Female	0.44 (0.39–0.49)***	0.27 (0.18–0.39)***	0.12 (0.10–0.13)***

No of persons in households			
1–2 persons	–		
3–4 persons	1.22 (0.99–1.50)*	–	–
5–9 persons	1.53 (1.25–1.88)***		
10 or more persons	2.38 (1.73–3.26)***		

Place of residence			
Urban	–	–	–
Rural	1.35 (1.18–1.53)***	1.11 (0.85–1.46)	1.04 (0.93–1.16)

Educational level			
No formal education	–	–	–
Less than primary	1.02 (0.86–1.21)	1.17 (0.75–1.80)	1.07 (0.92–1.24)
Primary completed	0.70 (0.59–0.83)***	1.12 (0.68–1.84)	0.97 (0.82–1.15)
Less than secondary	0.60 (0.50–0.72)***	0.94 (0.62–1.45)	1.05 (0.91–1.23)
Secondary & above	0.51 (0.42–0.63)***	0.56 (0.36–0.88)**	1.32 (1.1–1.5)***

Wealth index			
Lowest (1st quintile)	–	–	–
Low (2nd quintile)	0.84 (0.70–0.99)*	1.16 (0.70–1.93)	1.00 (0.86–1.16)
Middle (3rd quintile)	0.81 (0.68–0.98)**	1.27 (0.76–2.10)	1.18 (1.00–1.38)*
High (4th quintile)	0.75 (0.63–0.90)**	1.47 (0.88–2.46)	1.20 (1.11–1.41)**
Highest (5th quintile)	0.63 (0.50–0.78)***	1.90 (1.08–3.34)**	1.22 (1.00–1.48)**

GHK on ETS exposure			
Yes	–	–	–
No	1.26 (0.92–1.73)	2.67 (0.72–9.99)*	0.65 (0.47–0.89)***

Specific HK on ETS			
No knowledge	–	–	–
Some knowledge	0.78 (0.59–1.03)*	0.64 (0.27–1.50)	1.25 (0.97–1.62)*
Good knowledge	0.65 (0.53–0.76)**	0.54 (0.44–0.67)	1.64 (1.3–2.05)***

Attitude about ETS (at home)			
Smoking allowed	–	–	–
Not allowed	0.01 (0.01–0.02)***	0.81 (0.56–1.18)	0.85 (0.75–0.96)**
No rules or policy	0.13 (0.11–0.16)***	0.93 (0.62–1.34)	0.96 (0.84–1.08)

Attitude about ETS (at workplace)			
Smoking allowed	–	–	–
Not allowed		0.04 (0.02–0.05)***	
No rules or policy		0.34 (0.24–0.49)***	

Perception of SR at SP			
No supports			
Moderate supports	–	ns	–
Strong supports	0.90 (0.75–1.07)		3.58 (1.67–7.7)***
	0.73 (0.43–1.24)*		4.96 (2.4–10.3)***

GHK-general health knowledge, SR-smoking restrictions, SP-some places (workplaces, restaurants, universities, etc.), OR-odds ratio, CI-confidence interval, ns-not significant in bivariate analysis, *p < 0.05; **p < 0.01; ***p < 0.001, Model A: exposed at home, Model B: exposed at workplace, and Model C: exposed at public places.

General and specific health knowledge on ETS had significant influence on exposure at home. For instance, people with no general health knowledge on ETS had 1.3 times higher likelihood to be exposed at home. In addition, people with some knowledge (OR = 0.78, 95%CI = 0.59–1.03) and good knowledge (OR = 0.65, 95% CI = 0.74–1.19) had lower chance to be exposed ETS at home compared with people with no knowledge. Attitudes to ETS at home was significantly (p < 0.001) associated with lower exposure level. There was about 99% and 87% lower chance of ETS exposure if smoking was not allowed and no rules/policy at home compared to smoking allowed at home respectively. Perception about ETS also influenced exposure level at home. For example, moderate (OR = 0.90, 95% CI = 0.75–1.07) and strong supports (OR = 0.73, 95% CI = 0.43–1.24) of smoking restrictions in some places was associated with lower likelihood of ETS exposure at home.

For Model B, it was apparent that adults aged 25–44 years old had 1.44 times higher likelihood of ETS exposure at workplaces than reference category of 15–24 years old. Respondents aged 45 and above had less likelihood to be exposed at workplaces compare to reference group. Females had 73% less likelihood (p < 0.001) to be exposed at workplaces than their male counterparts. Exposure at workplaces did not show significant difference between urban and rural residents (OR = 1.11, 95% CI = 0.85–1.46). Respondents with higher education level (secondary & above) had 44% lower odds to be exposed than respondents with no education. The other educational categories did not show any significant differences with the reference category. Respondents with higher wealth index had higher likelihood to be exposed at workplaces. For example, people living with highest wealth quintile had 1.9 times higher likelihood to be exposed at workplaces than people living with lowest quintile.

General and specific health knowledge on ETS had significant influence on exposure at workplaces. For instance, people with no general knowledge on ETS had 2.7 times higher likelihood to be exposed at workplaces. In addition, people with some knowledge (OR = 0.64, 95% CI = 0.27–1.50) and good knowledge (OR = 0.54, 95% CI = 0.44–0.67) had lower odds of ETS exposure at home compared to people without knowledge. Attitudes to ETS at home was associated with lower likelihood of exposure at workplaces. There was 19% and 7% lower chance of ETS exposure at workplaces if smoking not allowed at home or no rules/policy compared smoking allowed at home respectively. In addition, attitudes to ETS at workplaces was significantly (p < 0.001) associated with lower exposure. There were 96% and 66% lower odds of exposure if smoking not allowed at workplaces and no rules/policy compared to smoking allowed respectively.

For Model C, adults aged 45–59 and 60+ years old had significantly (OR = 0.84, 95% CI = 0.72–0.97; OR = 0.43, 95% CI = 0.35–0.51) lower odds of ETS exposure at public places compared to reference category of 15–24 years old. Females were 88% less likely (p < 0.001) to be exposed at public places than their male counterparts. Exposure at public places did not show any difference between urban and rural residents (OR = 1.04, 95% CI = 0.93–1.16). Respondents with higher education level (secondary & above) had 1.3 times higher likelihood of ETS exposure at public places than respondents with no education. The other educational groups did not show any differences with reference category of no education. People with higher wealth index, had higher likelihood to be exposed at public places. For instance, people living in middle to highest wealth quintiles have more or less 1.2 times higher likelihood (p < 0.01 to p < 0.05) to be exposed at public places than people living in lowest wealth quintile.

In Bangladeshi communities, general and specific health knowledge on ETS exposure has negatively influenced ETS exposure in public places. For example, respondents with general health knowledge on ETS exposure has 35% higher chance (p < 0.001) to be exposed at public places than respondents without health knowledge of ETS exposure. Moreover, people with some knowledge (OR = 1.25, 95% CI = 0.97–1.62) and good knowledge (OR = 1.64, 95% CI = 1.31–2.05) about ETS exposure had significantly (p < 0.05 and p < 0.001) higher likelihood of ETS exposure at public places. Attitudes to ETS at home had positive impacts on exposure at public places. There was about 15% (p < 0.01) and 4% lower odds of exposure at public places if smoking not allowed at home or no rules/policy compared to smoking allowed at home respectively. Perception about ETS also influenced exposure level at public places but with different dimensions. For instance, moderate (OR = 3.6, 95% CI = 1.67–7.71) and strong supports (OR = 5.00, 95% CI = 2.40–10.30) of smoking restrictions in some places was associated with higher likelihood of ETS exposure (p < 0.001) at public places.

### 3.4 Association between TC and ETS exposure

In Model A, the respondents who smoked daily and less than daily had 2.8, and 1.5 times higher likelihood of exposed ETS at home compared with the reference category of no tobacco smoking. In addition, respondents who used any smokeless tobacco daily (OR = 1.90, 95% CI = 1.40–2.55) and less than daily (OR = 1.31, 95% CI = 1.13–1.50) were significantly (p < 0.001) at higher likelihood of ETS exposure at home than their non-user counterpart. In Model B, it was found that daily smoking of tobacco (OR = 1.32, 95% CI = 0.98–1.79) and smokeless tobacco (OR = 1.40, 95% CI = 0.99–1.98) were significantly associated with higher likelihood of ETS exposure at workplaces compared with the reference category of non-users. In Model C, the respondents who smoked daily and less than daily had 1.5 and 1.2 times higher likelihood of exposure to ETS at public places than their non-user counterpart. Besides, respondents who used any smokeless tobacco daily or less than daily were 1.4 times (p < 0.001) more likely to be exposed to ETS at public places compared with the reference category of non-users (Table 5).

**Table 5:** Association between ETS and TC exposure in different settings.

Factors	ETS exposure in different settings		
	Model A	Model B	Model C
	AOR (95% CI)	AOR (95% CI)	AOR (95% CI)
Currently ST			
No	–	–	–
Less than daily smoking	1.49 (1.39–1.90)**	1.21 (0.98–1.47)	1.19 (0.89–1.42)**
Daily smoking	2.83 (2.39–3.34)***	1.32 (0.98–1.79)*	1.53 (1.33–1.76)***

Currently using SLT			
No	–	–	–
Less than daily using	1.31 (1.13–1.50)***	1.24 (0.99–1.52)	1.35 (1.20–1.53)***
Daily using	1.89 (1.40–2.55)***	1.40 (0.99–1.98)*	1.38 (1.07–1.79)***

AOR-adjusted odds ratio, CI-confidence interval, *p < 0.05; **p < 0.01; ***p < 0.001, Model A: exposed at home, Model B: exposed at workplace, and Model C: exposed at public places, ST-smoked tobacco, SLT-smokeless tobacco.

## 4 Discussion

>50% of Bangladeshi adults were exposed ETS at home comprising more males than females. Adults were also exposed at workplaces with male dominance. The exposure level was higher in productive ages 25–59 years old. There are consistent with other studies (Abdullah et al., 2011; ITC, 2006). The ETS exposure was also high at public places. Males had overwhelmingly higher exposure at public places than females (Abdullah et al., 2011; Palipudi et al., 2011; Rachiotis et al., 2010; Rudatsikira et al., 2007). These lower levels of exposure among women compared to men in work and all the public places suggest that female who are mainly housewives and unemployed were less likely to visit these public places. Besides, adults aged 15–44 had the highest exposure level than other age categories. This is also consistent with ITC 2010 Survey findings. We found that respondents aged 45+ years old had significantly lower risk of ETS exposure at home. Similar findings were also reported in other empirical studies (Abdullah et al., 2011; Palipudi et al., 2011) where it was clear that age was significantly associated with ETS exposure at home. The higher the number of persons in a household, the higher the risk to be exposed to ETS at home. Similar findings were also reported in some studies (Akhtar et al., 2007; Bolte et al., 2009; Hyland et al., 2009; Sims et al., 2010) and this may be due to having more smokers in the household. In line with other studies (Abdullah et al., 2011; Palipudi et al., 2011) place of residence also showed significant associations. For instance, rural respondents had significantly higher risk to be exposed to ETS than urban counterparts and this may be also related to knowledge gap. The low SES population had higher rates of smoking and thus a higher likelihood of exposure to ETS (Abdullah et al., 2011).

Mixed results were found in Bangladesh in general and specific health knowledge about ETS. For instance, people in Bangladesh with some knowledge and good knowledge had lower likelihood to be exposed to ETS at home compared with people without any knowledge. In contrast with Bangladeshi adults, Indian adults with some knowledge and good knowledge had higher likelihood to be exposed to ETS at home. This may be due to socio-economic and cultural differences (Abdullah, Yang, Beard, & Cao, 2010; Chen et al., 2009; Liu et al., 2008; Mak et al., 2008; Mei et al., 2009; Oberg et al., 2011). We found that, attitudes to ETS at home were significantly associated with lower exposure level and support for smoke-free homes. This is consistent with other studies (Abdullah et al., 2010; Chen et al., 2009; Liu et al., 2008; Mak et al., 2008; Mei et al., 2009; Oberg et al., 2011) where awareness of health risks of ETS through knowledge, attitude and perception towards ETS and higher education was positively associated with support for smoke-free homes.

Like ETS exposure at home, respondents were also exposed to ETS at workplace. Consistent with the findings of (Abdullah et al., 2011; Palipudi et al., 2011), Bangladeshi adults aged 25–44 years old were more likely to be exposed to ETS at workplace than younger ones (15–24 years old). This may be due to productive age groups and working status. Females were less likelihood to be exposed at workplace than their male counterparts. This may be due to gender gap in employment. In South-Asian region females are mostly housewives and engaged in self-employment (World Health Organization, 2011a, World Health Organization, 2011b). The urban-rural differences in exposure level at workplace were not substantial. This was also found insignificant in (Palipudi et al., 2011). Education may be a positive influence where illiterate smokers and non-smokers might not fully understand the health risks of ETS (Abdullah et al., 2011; Palipudi et al., 2011). Higher education was positively associated with support for smoke-free workplace (Chen et al., 2009; Liu et al., 2008; Mak et al., 2008; Oberg et al., 2011). Consistent with other findings, this study showed respondents with higher education in Bangladesh to have significantly lower risk to be exposed to ETS at workplace than respondents with no education at all. In contrast with other studies (Abdullah et al., 2011), Bangladeshi adults have the higher likelihood to be exposed at workplace if they come from higher wealth quintiles. This may be due to working environment.

Awareness of health risks of ETS through knowledge, attitude and perception towards ETS was positively associated with support for smoke-free workplaces (Chen et al., 2009; Liu et al., 2008; Mei et al., 2009; Oberg et al., 2011). It was found that general and specific health knowledge on ETS had significantly influenced the exposure at workplace. For instance, an inverse association was found between knowledge about adverse effects of ETS and risk of ETS exposure at workplace. Knowledge of health risks and attitude towards smoking were associated with supporting smoking restrictions and quitting (Abdullah et al., 2010; Chen et al., 2009; Lim et al., 2006; Mei et al., 2009). We found that attitudes to ETS at home were associated with lower likelihood of exposure at workplace. In addition, attitudes to ETS at workplace also had significant inverse association with ETS exposure level in Bangladesh.

Older people (45 years and above) and females were less likely to be exposed to ETS at public places. This might be due less time spend outside home. These findings were consistent with other studies (Akhtar et al., 2007; Bolte et al., 2009; Hyland et al., 2009; Sims et al., 2010). Interestingly, respondents with higher education level (secondary & above) had higher likelihood of exposure ETS at public places than respondents with no education. This might be because educated people were more responsive about the exposure level than their non-educated counterpart. A qualitative research is therefore suggested in this regard. Mixed results were found in the case of asset quintiles and ETS exposure and need further investigations. Awareness of health risks of ETS through knowledge, attitude and perception towards ETS was positively associated with support for smoke-free public places (Chen et al., 2009; Liu et al., 2008; Mei et al., 2009; Oberg et al., 2011). Consistent with other findings, this study showed, general and specific health knowledge on ETS exposure significantly influenced the exposure in public places. This might be because people with good knowledge about the adverse effects of ETS are more responsive than their counterpart without any knowledge.

In Bangladesh, there is diverse and frequent use of smoking tobacco products other than cigarettes like *bidis*, *kreteks*, cheroots, hookah etc., crowding, lack of awareness at public places and workplaces, and weak or little enforcement of legal provisions to protect those exposed to ETS (World Health Organization, 2009a, World Health Organization, 2009b). Enforcement of smoke-free laws in Bangladesh is weak but this is improving as stronger legislation is enacted, rigorous enforcement is demanded by people who have growing awareness on the harms of ETS exposure (Oberg et al., 2011). These policies contribute decisively to smoking reduction, and help with the approval and implementation of other policies that reduce tobacco demand, such as a comprehensive ban of tobacco advertisement, promotion, and sponsorship. Making policies for 100% smoke-free environment is the most effective way to protect the public, including children, women, and people at their homes, workplaces, and public places from exposure to ETS. There is sufficient evidence that implementation of smoke free policies substantially decreases ETS exposure (Oberg et al., 2011; Pierce & Leon, 2008). Studies of the effects of the smoke-free policies consistently show that these policies decrease exposure to ETS by 80–90% in high exposure settings, and they can lead to overall decreases in exposure of up to 40% (Haw & Gruer, 2007). Some special techniques such as unannounced inspections, surprise checks and raids by the empowered government agency can be very effective deterrents for erring public places. To this end, people must be made aware about their rights to demand clean, tobacco-free air in public places as well as in workplaces. Increased awareness of the considerable health risks posed by ETS at home, workplaces and public places and concerns for public safety have led to an active movement to impose a total ban on smoking at public places. The results clearly indicate that smoke-free policy needs to be strengthened by declaring more and more public places 100% smoke free in Bangladesh. The study also suggests that innovative programs are needed to enhance the implementation of home smoking bans and workplace as well. Awareness campaigns through effective public education, media advocacy and communication are the key to implement smoke-free policies. Government and communities need to work together to create smoke-free environments. Appropriate strategies need to be developed to involve the private sector and communities to ensure success for the campaign for smoke-free environments.

### 4.1 Study limitations and future directions

Self-reported data on ETS could suffer from recall bias and deliberate misreporting. This misreporting could influence the prevalence, patterns, and determinants of ETS. Although many variables were analysed, exclusion of some other variables might limit the findings. Finally, since the datasets were cross-sectional, cause-effect relationships could not be inferred.

To ensure valid data for reliable findings, verifications through biomarkers such as cotinine assessment or exhaled carbon monoxide or saliva, urine, blood and hair could be used in national surveys. Along with these, the assessment of ETS exposure can measure indoor air concentrations of ETS constituents, and through constant monitoring. The country has the task forces to implement smoke-free policies which need to be further strengthened and functioning in collaboration with community participation. Bangladesh has enacted tobacco control legislation, laws and policies to protect people from ETS exposure. However, most of these existing measures are partial and inadequate, and do not provide for a complete ban on smoking at public places. Further, the level of implementation and enforcement varies across national and sub-national levels. Despite all public places being declared smoke-free, compliance levels are not consistent. As a result, diligent implementation of provisions of the law, backed by compliance studies and public opinion polls that inform policy makers, public and the media play a crucial role in initiating and maintaining smoke-free efforts. The most important challenge so far has been effective enforcement of smoke-free laws. The mechanism of enforcement of laws is not well spread at the grassroots levels but mostly in urban-centric and limited to selected few metropolitans and large cities. The benefits of going smoke-free have not reached the people living in semi-urban and rural areas. Legislations must mandate implementation of complete smoke-free environments, as opposed to voluntary policies, in order to protect public health. Longitudinal surveys and cohort studies are recommended for examining the issue. Future studies that are qualitative in nature would also be useful for a better understanding of ETS exposure patterns towards effective public health control.

## Footnotes

1 Consist of a small amount of crushed tobacco, hand-wrapped in dried *temburni or tendu* leaves, and tied with string. It tends to deliver more tar and carbon monoxide than manufactured cigarettes because users must puff harder to keep them lit. *Bidis* are mostly used in South Asian countries.
